# Simulation based evaluation of cardiac motion estimation methods in tagged-MR Image sequences

**DOI:** 10.1186/1532-429X-13-S1-P360

**Published:** 2011-02-02

**Authors:** Patrick Clarysse, Jean Tafazzoli, Philippe Delachartre, Pierre Croisille

**Affiliations:** 1 CREATIS,CNRS UMR 5220,InsermU630, Villeurbanne, France

## Introduction

Several methods have been proposed to estimate myocardial motion from those based on the prior extraction of the tagging pattern to methods based on spatial phases [1]. Among several, the HARP method is one of the most popular methods of the latter group. Recently, an alternative method has been proposed: SinMod. It is however very difficult to objectively compare the accuracy of the methods.

## Purpose

The objective of this paper is to propose a methodology to evaluate concurrently methods for estimating motion displacement fields from 2D tagged-MR image sequences.

## Methods

We propose to evaluate the accuracy of the methods on realistic simulated tagged-MRI sequences. A simple kinematic mode based model of the heart deformation in the small axis plane continuously warps a true tagged-MR image at end-diastole to provide a whole cycle simulated sequence. Various conditions can be varied from the models’ parameters to additive image noise, leading to a set of simulate sequences with various properties. Five sequences were simulated with increasing motion complexity from simple thickening (30%), rotation (10,20deg) to more realistic deformations without and with local anomaly. Then, estimation was carried out with the SinMod [1] (integrated into inTag OsiriX plugin) method. Thanks to the known motion, error indices were quantified between estimated and true dense motion fields as point wise displacement orientation and magnitude discrepancies. Global as well as phase by phase error statistics are delivered through error images and box and whiskers graphs.

## Results

With the thickening sequence, mean magnitude error is less than 0.5 pixels for all the phases. Angular shift error is in average less than 4deg with equivalent deviation. For the sequence with 10deg rotation, mean magnitude and angular errors remain less than 0.5pix and 8deg (median), respectively. Generally, errors are higher at end-systole. These values are significantly higher with the 20deg rotation sequence with up to 24deg and 8pix at late phases due to very large displacements into the myocardium. For the realistic sequences, the median magnitude error is systematically less than 1pix but angular shift reached 16deg. Similar results are obtained with the pathological case.

## Conclusions

This simulation based approach allows to finely compare method’s accuracy and highlight their respective strengths and weaknesses.
